# Clinical Evaluation of Low-Dose Magnesium Carbonate as a Phosphate Binder in Chronic Hemodialysis Patients

**DOI:** 10.3390/life16071213

**Published:** 2026-07-22

**Authors:** Valeri Tzekov, Tanya Kostadinova, Elizabet Artinyan, Rumyana Stoyanova, Evelina Valcheva, Nikolay Dimov

**Affiliations:** 1 Second Department of Internal Diseases, Section of Nephrology, Medical University of Plovdiv, 15A Vasil Aprilov Blvd, 4002 Plovdiv, Bulgaria; valeri.tsekov@mu-plovdiv.bg; 2 First Dialysis Services Bulgaria—Dialysis Center, 4002 Plovdiv, Bulgaria; kostadinova.tania@gmail.com (T.K.); dr.elizabeth.artiinyan@gmail.com (E.A.); 3 Clinic of Nephrology, University Multiprofile Hospital for Active Treatment “Sv. Georgi”, Plovdiv, 15A Vasil Aprilov Blvd, 4002 Plovdiv, Bulgaria; evelinavalcheva98@gmail.com; 4 Department of Health Management and Health Economics, Faculty of Public Health, Medical University of Plovdiv, 15A Vasil Aprilov Blvd, 4002 Plovdiv, Bulgaria; rumi_stoqnova@abv.bg

**Keywords:** CKD, hemodialysis, hyperphosphatemia, magnesium-based phosphate binders

## Abstract

**Background and Objectives:** Hyperphosphatemia is a key component of chronic kidney disease-mineral and bone disorder and is associated with higher rates of cardiovascular events and mortality in patients undergoing dialysis. Phosphate binders are essential for phosphorus reduction, and their evaluation relies on several factors, such as chemical composition, binding capacity, and safety profile. Unfortunately, long-term management is hindered by high pill burden and poor adherence. Magnesium phosphate binders have proven effectiveness in reducing phosphate levels. Nevertheless, despite their efficacy, they are not widely used in clinical settings because of concerns related to their use. This study assessed the efficacy of low-dose magnesium carbonate as a phosphate binder in patients undergoing chronic dialysis, focusing on its biochemical control, tolerability, and cost-effectiveness. **Materials and Methods:** A prospective observational study was conducted on 54 hemodialysis patients with end-stage renal disease at a single dialysis center. Patients were taking either 250 mg magnesium carbonate or 2400 mg sevelamer carbonate for 3 months. **Results:** Both groups showed decreased phosphorus levels, with a 14.2% significant reduction in the magnesium carbonate group (*p* < 0.001) and a 5.1% reduction in the sevelamer carbonate group. At the end of the study, no significant differences were observed between the groups (*p* = 0.682). In the magnesium carbonate group at month 3, compared to baseline, no significant differences were detected in other laboratory parameters reflecting calcium–phosphorus metabolism (Ca—*p* = 0.681, PTH—*p* = 0.126). Simultaneously, good compliance without clinically relevant gastrointestinal side effects was observed, including the absence of clinically significant hypermagnesemia. **Conclusions:** The phosphorus-lowering potential of low-dose magnesium carbonate is non-inferior to that of low-dose sevelamer carbonate. However, its favorable safety profile, low incidence of adverse effects, and cost-effectiveness make it a promising option in clinical practice, further highlighting the need for better recognition by physicians.

## 1. Introduction

Phosphate is an essential electrolyte that supports energy generation, muscle activity, and mineral metabolism. Most absorbed phosphate is retained in the skeleton with calcium, contributing to bone mineralization and integrity [1]. Bone and mineral metabolism are regulated by parathyroid hormone (PTH), vitamin D, fibroblast growth factor 23 (FGF23), and calcitonin via the bones, kidneys, and gastrointestinal system.

In patients with chronic kidney disease (CKD), both calcium and phosphate metabolism are impaired. These calcium-phosphorus abnormalities are termed Chronic Kidney Disease-Mineral Bone Disorder (CKD-MBD) [2]. The risk of mineral and bone disturbances increases from stage 3a onward and worsens through stages 4 and 5, peaking in patients undergoing hemodialysis [3].

An important aspect of CKD-MBD treatment is the management of hyperphosphatemia. Hyperphosphatemia is an independent predictor of cardiovascular disease and mortality in patients with CKD [4,5]. For patients undergoing hemodialysis, hyperphosphatemia is treated by severely restricting dietary phosphorus intake, using phosphate binders, and enhancing phosphorus elimination via dialysis. 

Phosphate binders remain a key treatment tool for CKD-MBD. Phosphate binders are classified as follows: calcium-based binders, which are commonly used but may cause calcium overload and vascular calcification; aluminum-based binders, which are effective but rarely used long-term because of toxicity risks; calcium-free, aluminum-free binders (sevelamer hydrochloride/carbonate, lanthanum carbonate), which are safer alternatives without calcium- or aluminum-related side effects but have higher costs. Iron- and magnesium-based binders are also available [6,7,8,9]. There are certain characteristics that a phosphate binder must possess in order to be considered as “an ideal.” These include high affinity for phosphate binding and effectiveness at lower doses (and therefore low pill burden), rapid binding of phosphate irrespective of ambient pH, low solubility, minimal to no systemic absorption, non-toxicity and absence of side effects, availability as a solid oral dosage form, acceptable palatability to promote adherence, and low cost [10,11]. Over the years, magnesium salts have attracted interest because of their favorable safety profiles and cost-effectiveness. However, the clinical evidence remains less extensive than that for more established binders, highlighting the need for further research.

Interestingly, studies directly comparing the overall effectiveness of magnesium binder regimens with that of sevelamer formulations are lacking.

This study aimed to evaluate the effectiveness of low doses of magnesium carbonate compared with low doses of sevelamer carbonate as a phosphate binder in patients undergoing chronic dialysis.

## 2. Materials and Methods

### 2.1. Study Design 

A prospective observational pilot study was conducted in patients undergoing hemodialysis for end-stage renal disease (ESRD) treated in a single dialysis center in Plovdiv, Bulgaria (First Dialysis Services Bulgaria EAD). The protocol for this study was approved by the Ethics Committee of First Dialysis Services Bulgaria EAD. 

### 2.2. Eligibility Criteria

#### 2.2.1. Inclusion Criteria

The inclusion criteria were as follows: signed informed consent; age over 18 years; undergoing dialysis for more than 1 year, without residual renal function; and hyperphosphatemia with serum phosphorus levels > 1.8 mmol/L (>5.5 mg/dL) on at least two consecutive monthly measurements. 

#### 2.2.2. Exclusion Criteria

The exclusion criteria were as follows: normophosphatemia or phosphorus levels < 1.8 mmol/L; prior parathyroidectomy; treatment with calcimimetics or active vitamin D analogs within the 3 months preceding enrolment; psychiatric or cognitive disorders likely to impair adherence; and any acute intercurrent illness at baseline.

### 2.3. Study Protocol and Participants

All patients meeting the eligibility criteria were given the opportunity to choose whether to be treated with sevelamer carbonate or magnesium carbonate. According to their choice, the patients were assigned to two groups. Patients in the first group received magnesium carbonate at a dose of one tablet daily. Each tablet contained 250 mg of magnesium carbonate, which is equivalent to 71 mg of elemental magnesium. 

Patients in the second group received sevelamer carbonate at a dose of three tablets daily (2400 mg). Each tablet of the medication contains 800 mg of sevelamer carbonate.

After the start of the study and throughout its entire duration, all patients received high-flux hemodialysis three times per week for 4 h per session with bicarbonate dialysate containing magnesium 0.50 mmol/L and calcium 1.5 mmol/L. The Kt/Vurea values in the patients ranged between 1.4 and 1.6 according to the Kidney Disease: Improving Global Outcomes (KDIGO) criteria [12]. 

No other oral phosphate binders, or vitamin D or calcimimetics, were co-prescribed during the study. Patients were given recommendations to follow a diet low in phosphorus. The patients were monitored for three months.

### 2.4. Sample Collection and Follow-Up

Fasting pre-dialysis blood samples were obtained from the arterial dialysis line at baseline and monthly for 3 months (months 1, 2, and 3). At baseline, complete blood count, routine biochemical parameters, electrolytes, and intact PTH were assessed. During follow-up, the levels of calcium, magnesium and phosphorus were measured monthly, whereas PTH was obtained at baseline and at the 3rd month of treatment in accordance with KDIGO recommendations. 

### 2.5. Outcomes

Using the concept of an ideal phosphate binder, we predefined primary, secondary, and tertiary outcomes to evaluate their effectiveness. 

The primary outcome was the change in serum phosphorus from baseline to month 3 in each treatment group and the proportion of patients achieving the KDOQI target range (1.13–1.78 mmol/L).

Secondary outcomes included the evaluation of parameters related to CKD-MBD, specifically serum calcium (Ca) levels, PTH levels, and the Ca × P product (Ca:P ratio).

Adverse effects (gastrointestinal irritability, allergic reactions, and hypermagnesemia), pill burden, and cost–benefit analyses constituted the tertiary criteria. 

Tertiary outcomes included: (i) occurrence of adverse events, particularly gastrointestinal symptoms and clinical manifestations compatible with hypermagnesemia (assessed using patient-completed questionnaire cards); (ii) biochemical hypermagnesemia, defined as serum magnesium > 1.06 mmol/L according to the local laboratory reference range; (iii) pill burden, expressed as the number of phosphate binder tablets per day; and (iv) direct drug cost per day, month, and year, evaluated by comparing prices according to the current pricing policy.

### 2.6. Statistical Analysis

Descriptive statistics and parametric tests were used for data analysis. Continuous variables are presented as means ± standard deviations (SDs) and categorical variables as counts and percentages. Within-group changes from baseline to month 3 were assessed using paired *t*-tests for normally distributed variables. Between-group comparisons at baseline and at each follow-up time point were performed using independent-samples *t*-tests. To evaluate whether the means of a dependent variable are equal across levels of one or more categorical independent variables and across one or more continuous variables, ANCOVA was used.

A significance level of *p* < 0.05 was set for the null hypothesis. SPSS version 23 and MS Excel 2016 software were utilized for data processing.

## 3. Results

### 3.1. Demographic Characteristics 

A total of 60 patients were enrolled in this study. Six patients dropped out, while the remaining 54 completed the study; 53.7% (*n* = 29) were assigned to the magnesium carbonate group and 46.3% (*n* = 25) to the sevelamer carbonate group. The baseline laboratory parameters and demographic characteristics of both groups are presented in Table 1.

In terms of demographic parameters, no statistically significant differences were observed in age (*p* = 0.105) or sex (*p* = 0.600) between the two patient groups. Regarding the laboratory parameters related to CKD-MBD, the mean phosphorus levels were higher in the magnesium carbonate group than in the sevelamer carbonate group, whereas the mean calcium levels were higher in the sevelamer group. Ultimately, no statistically significant differences were observed between the two parameters. Meanwhile, magnesium and PTH levels were higher in the sevelamer group, and statistically significant differences were found for both parameters. No statistically significant differences were found between the two groups for the remaining laboratory parameters.

### 3.2. Primary and Secondary Criteria

**Figure 1:**
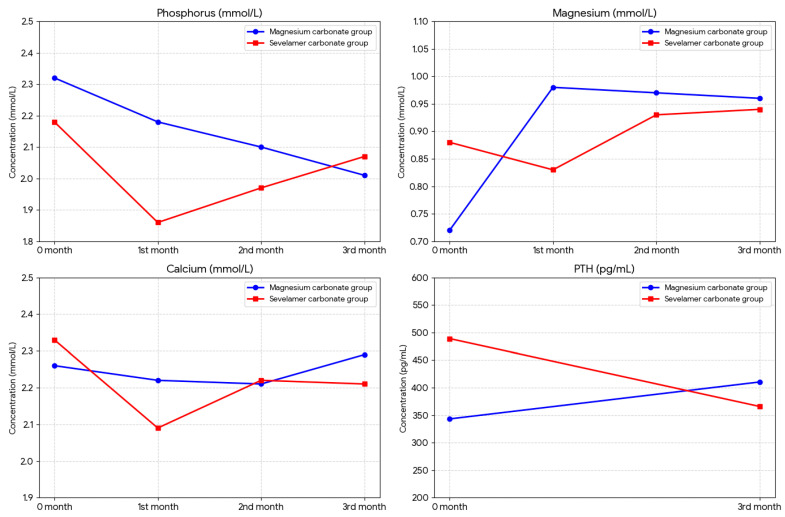
Comparison of the changes in the main CKD-MBD parameters over time in the two observed groups: The magnesium carbonate group and the sevelamer carbonate group.

The main laboratory parameters monitored over time in both groups are shown in Figure 1. Before treatment initiation, the mean phosphorus level in the magnesium carbonate group was 2.30 ± 0.35 mmol/L. Over the subsequent months, a decrease in mean phosphorus levels was observed, reaching 2.01 ± 0.45 mmol/L at the 3rd month. The mean calcium levels remained relatively stable throughout the study period, whereas the mean PTH levels showed an increasing trend.

At the same time, the mean serum phosphorus level in the sevelamer carbonate group before the initiation of therapy was 2.18 ± 0.37 mmol/L. A decreasing trend in phosphorus levels was observed at different time points. Concurrently, a reduction in the mean serum calcium and PTH levels was recorded at the respective time points over the study period.

A paired *t*-test was performed to compare the mean values of the parameters at baseline and at month 3 after initiation of therapy within both groups. In the magnesium carbonate group, phosphorus levels decreased significantly, with a mean reduction of 0.327 mmol/L (t = 4.926, df = 27, *p* < 0.001). Calcium levels remained relatively stable and PTH changes were not statistically significant as well (Table 2).

In the sevelamer carbonate group, no statistically significant differences were detected in serum phosphorus and PTH levels. In contrast, a statistically significant change was observed in mean serum calcium levels between baseline and month 3 (Table 3).

**Figure 2:**
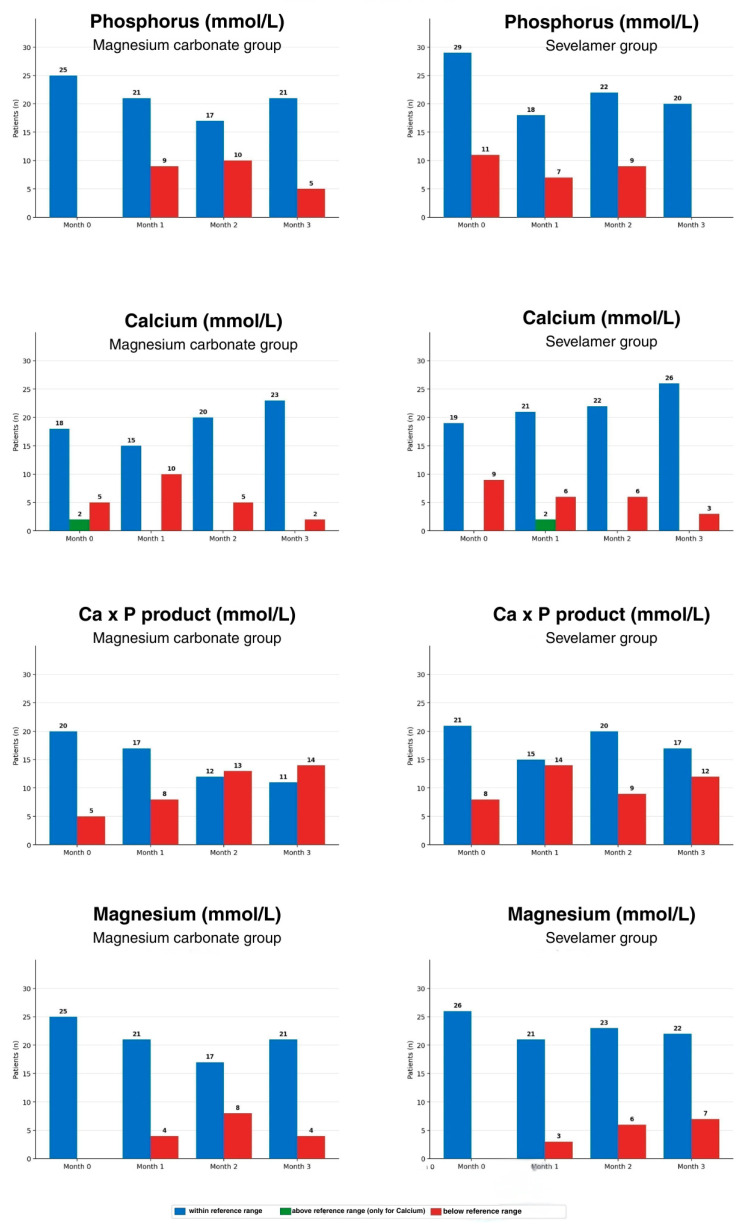
Comparison of the number of patients in the two groups within reference ranges, within normal limits, and outside normal limits for the monitored parameters by month. The reference ranges for each parameter are as follows: Phosphorus (mmol/L): <1.8 and >1.8; Calcium (mmol/L): 2.12–2.64; Ca × P product: <4.4 and >4.4; Mg (mmol/L): 0.73–1.06.

Figure 2 compares the number of patients in the two groups within reference ranges/within normal limits, and outside normal limits for the monitored parameters at each month. In the magnesium carbonate group, all patients had serum phosphorus levels > 1.8 mmol/L at baseline. Over the course of follow-up, the number of patients with serum phosphorus levels > 1.8 mmol/L progressively declined, and by month 3, nine (31.0%) patients had achieved target phosphorus levels according to the KDOQI criteria.

For serum calcium, 10 patients had values outside the reference range at baseline, whereas nearly 90% (*n* = 26) had serum calcium within the reference interval by month 3. Meanwhile, 72.4% (*n* = 21) of patients initially exhibited an abnormal Ca × P product (>4.4). By month 3, a reduction in the proportion of patients with an elevated Ca × P product was observed, with 41.3% (*n* = 12) falling within the target range for this parameter.

Similarly, regarding phosphorus levels in the sevelamer carbonate group, all patients had serum phosphorus levels > 1.8 mmol/L at baseline. Over the follow-up period, the number of patients with serum phosphorus levels > 1.8 mmol/L declined, and by month 3, five (20.0%) patients had achieved target phosphorus levels according to the KDOQI criteria. For serum calcium, seven patients had values outside the reference range at baseline, whereas by the 3rd month, 92% (*n* = 23) of patients had serum calcium within the reference interval. Concurrently, 80.0% (*n* = 20) of the patients initially exhibited an abnormal Ca × P product (>4.4). By month 3, a reduction in the proportion of patients with an elevated Ca × P product was observed, with 56.0% (*n* = 14) falling within the target range.

Table 4 presents the results of independent samples *t*-tests comparing the primary parameters between the two groups at months 1, 2, and 3. No statistically significant differences were found in mean serum magnesium levels at months 1, 2, and 3, or in mean PTH levels at month 3 between the two study groups. A statistically significant difference was noted in mean phosphorus (*p* = 0.010) and calcium (*p* = 0.035) levels at month 1 of treatment, although at the end, there was no recorded difference for all parameters—Ca, P, Mg or PTH. To evaluate the comparative efficacy of the magnesium- versus sevelamer-based regimen, a longitudinal analysis of covariance (ANCOVA) was conducted. At baseline, there was a statistically significant difference between the two groups in terms of serum magnesium and PTH, with the mean values of both parameters being higher in patients in the sevelamer carbonate group. After adjusting for the initial baseline magnesium deficit, the magnesium cohort achieved significantly higher adjusted serum magnesium levels than the sevelamer group at month 2 (F(1,51) = 13.13, *p* < 0.001) and month 3 (F(1,50) = 7.98, *p* = 0.007). For PTH at month 3, the unadjusted independent-samples *t*-test showed no significant difference between treatment groups (t(51) = 0.41, *p* = 0.682). When adjusting for baseline PTH (PTH_0), the ANCOVA was not statistically significant under either the weak correlation assumption ((r = 0.3), F(1,50) = 0.91, *p* = 0.345) or the moderate correlation assumption ((r = 0.5), F(1, 50) = 2.08, *p* = 0.155). However, under a strong correlation (r = 0.7), sevelamer carbonate was associated with lower adjusted mean PTH values (294.711 ± 57.589 pg/mL) compared with magnesium carbonate (473.822 ± 54.324 pg/mL), with the overall model indicating statistical significance (F(1,50) = 4.97, *p* = 0.030).

### 3.3. Tertiary Outcomes

In the magnesium carbonate group, an increase in mean serum magnesium levels was observed at month 1 compared with baseline. In the following months, the mean values of this parameter remained stable, although a statistically significant increase was observed between baseline levels and those at month 3 of treatment (Figure 1). At the same time, the number of patients with abnormally elevated serum magnesium levels increased by only four over the entire study period (Table 4). Meanwhile, in the sevelamer group, mean serum magnesium levels were higher at all follow-up time points compared to baseline (Table 5). A paired *t*-test demonstrated an increase in serum magnesium of 0.063 mmol/L at month 3 (t = −3.097, *p* = 0.005) (Table 3). 

Over the entire observation period, the number of patients with abnormally elevated serum magnesium levels in both groups increased by only four individuals (Figure 2). No statistically significant between-group differences in mean serum magnesium levels were detected at months 1, 2, or 3 (Figure 1).

Regarding emerging adverse reactions, within the magnesium carbonate group, one patient reported nausea, and within the sevelamer group, one patient reported diarrhea. Both complications were managed symptomatically and did not lead to discontinuation of therapy. Neither group discontinued therapy as a result of these adverse effects.

Table 5 compares the costs of sevelamer carbonate and magnesium carbonate formulations available in our country at the time the study was conducted, including adverse effects and economic burden.

## 4. Discussion

The latest clinical guidelines recommend maintaining serum phosphate near the normal range, with KDOQI guidelines targeting 3.5–5.5 mg/dL in patients undergoing dialysis, while KDIGO guidelines recommend targeting phosphate “towards normal” levels [13,14]. However, achieving these targets remains problematic; contemporary data show that over 40% of dialysis patients have serum phosphate levels exceeding 5.5 mg/dL at any given month, and 77% exceed this threshold over a 6-month evaluation period [14]. 

The actual phosphorus removal by standard dialysis sessions lasting 4 h three times a week can range from 600 to 1200 mg per treatment [15]. As dietary restriction and dialysis alone often achieve only partial control of serum phosphorus levels, the use of specific medications (phosphate binders) is required.

The use of oral phosphate binders is associated with improved outcomes and survival, with calcium- and sevelamer-based binders being the most widely used ones. Magnesium-containing binders, whether used alone or in combination, have been proposed as a promising option for maintaining effective phosphate levels. Currently, only a few trials have examined combination regimens of magnesium salts with calcium or iron binders [16,17,18,19]. Nevertheless, evidence regarding the use of magnesium salts as monotherapy remains limited, and studies comparing magnesium with sevelamer are virtually absent. Consequently, in the present study, we compared magnesium carbonate with sevelamer carbonate using parameters aligned with the concept of an ideal phosphate binder.

**Efficacy of low-dose magnesium carbonate for reducing serum phosphorus**

The CALMAG study established the role of magnesium-based binders in clinical practice. This multicenter, randomized controlled trial of 255 hemodialysis patients demonstrated that calcium acetate/magnesium carbonate (CaMg) was non-inferior to sevelamer hydrochloride in lowering serum phosphorus levels to KDOQI targets after 24 weeks [18]. The mean serum phosphorus reduction was approximately 31% with CaMg and 29% with sevelamer hydrochloride over 25 weeks of treatment.

In another placebo-controlled study (Fermagate), an iron/magnesium combination was evaluated in 63 patients who took the medication three times daily for 21 days, and a reduction in serum phosphorus was observed [20]. Notably, patients in these studies received combination therapy with two phosphate binders. In most cases, magnesium salts are introduced to enhance the effect of another phosphate binder and/or to permit dose reduction of the latter in the presence of significant adverse effects. To date, magnesium carbonate used as monotherapy has only been compared with calcium acetate, and it has been demonstrated that magnesium carbonate achieved phosphorus control that was at least comparable to calcium acetate (serum phosphorus within KDOQI targets in 70.6% vs. 62.5% of patients, respectively) [21]. Surprisingly, no clinical trials have directly compared pure magnesium carbonate salts with sevelamer formulations to date, which was the rationale for the present comparative study.

In the present study, baseline mean serum phosphorus levels did not differ significantly between the two groups, although the values were numerically higher in the magnesium carbonate group. During the three-month treatment period, serum phosphorus levels decreased in both groups, with a statistically significant reduction in the magnesium carbonate group. The absolute phosphorus reduction in the magnesium carbonate group (0.33 mmol/L) was approximately 2.8 times greater than that in the sevelamer carbonate group (0.11 mmol/L). By the 3rd month, mean phosphorus levels were comparable between magnesium carbonate and sevelamer carbonate. Similarly, the proportion of patients achieving the KDOQI target serum phosphorus level was similar (30% in the magnesium carbonate group and 20% in the sevelamer carbonate group). Collectively, these findings indicate that magnesium carbonate is non-inferior to sevelamer carbonate with respect to serum phosphorus reduction over the study period.

**Evaluation of biochemical parameters related to CKD-MBD—serum calcium, PTH levels, and the Ca × P product**

Monitoring serum calcium levels is an important element in the overall assessment of patients undergoing dialysis. The KDIGO recommends that they should be kept within the normal reference range to avoid hypercalcemia [12]. Meta-analyses have shown a lower prevalence of hypercalcemia with sevelamer than with calcium-based binders [22,23]. Sevelamer carbonate typically does not increase serum calcium levels and may even slightly reduce them during treatment for hyperphosphatemia in CKD patients on dialysis [24,25].

In a study on treatment with combined calcium and magnesium carbonate formulations, it was reported that although total serum calcium increased slightly, ionized calcium levels remained stable, and the risk of hypercalcemia did not increase compared with sevelamer [18]. Meanwhile, studies evaluating the use of magnesium carbonate as monotherapy have reported that calcium levels were not altered [26,27].

In the current study, similar to previous studies, the calcium concentrations in the magnesium carbonate group remained relatively stable over time, with minimal intergroup variability across monthly assessments. The patients in the sevelamer group exhibited greater variability in their calcium levels. For instance, the mean baseline calcium level was higher in the sevelamer group and three months afterwards, it was lower than that in the magnesium carbonate group, reaching statistical significance. However, no statistically significant differences in serum calcium levels were observed between the two groups at baseline or after three months of therapy. Additionally, the proportion of patients with calcium values within the reference range was similar in both groups (approximately 90%).

Regarding the Ca × P product, both medications reduced the number of patients with abnormally elevated values; however, sevelamer carbonate demonstrated a modest advantage in terms of the proportion of patients reaching optimal Ca × P product levels. The results are consistent with other studies, with an approximately 25–30% reduction in the Ca × P product in the sevelamer group [22,28].

Changes in parathyroid hormone levels can influence the selection of the optimal phosphate binder. The use of these agents generally improves calcium–phosphorus homeostasis and, in most cases, reduces PTH levels. Sevelamer therapy is typically associated with decreased PTH and FGF-23 levels [29,30]. A similar pattern has been reported with magnesium carbonate, where previous studies have demonstrated reductions in PTH with monotherapy and in combination with other phosphate binders [19,27]. Notably, the CALMAG trial demonstrated an equivalent reduction in FGF-23 with the calcium acetate/magnesium carbonate combination, suggesting that this effect is primarily mediated by the degree of phosphate control achieved rather than by the specific binder used. In our study, a significant reduction in PTH levels was observed in patients treated with sevelamer; however, unexpectedly, PTH levels increased in the magnesium carbonate group. To understand this unexpected finding, we conducted an in-depth literature review of the impact of serum magnesium on PTH regulation. Hypomagnesemia impairs calcium-sensing receptor function and can paradoxically increase PTH secretion despite normocalcemia, whereas elevated magnesium enhances calcium-sensing receptor sensitivity and suppresses PTH release [31]. When used as a phosphate binder, magnesium carbonate raises serum magnesium concentrations and is thus expected to attenuate PTH secretion. In the study by Tzanakis, a total of 1552 mg of magnesium carbonate per day (6.2 tablets, range 3–9) was used, which led to a slight increase in serum magnesium from 2.38 to 2.59 mg/dL and a moderate decrease in PTH from 316 to 251 pg/mL (an approximate reduction of about 20%) [26]. In contrast, in our study, we hypothesized that the low doses of magnesium carbonate used were insufficient to produce a clinically significant rise in serum magnesium levels and, therefore, most likely led to an inability to suppress this feedback via the aforementioned pathophysiological mechanism. Therefore, we acknowledge that the size of the current patient cohort may serve as a potential limitation. Accordingly, future studies with larger cohorts should investigate the serum magnesium thresholds at which the use of magnesium as a phosphate binder results in PTH suppression.

**Tertiary Criteria: Adverse Effects, Pill Burden, and Cost–Benefit Analyses**

One of the principal reasons many dialysis centers avoid using magnesium carbonate is the concern about hypermagnesemia; accordingly, this parameter is of particular importance in the present study. Serum magnesium constitutes approximately 1% of the total body magnesium, which is predominantly intracellular [32]. The ionized fraction (approximately 60% of total serum magnesium) may be lower in patients undergoing hemodialysis than in individuals with preserved renal function, potentially leading to an overestimation of the risk of hypermagnesemia [33]. Hypermagnesemia is generally well tolerated, with concentrations of 1.05–2.2 mmol/L (2.55–5.35 mg/dL) being asymptomatic. When the levels reach 2.2–3.5 mmol/L, symptoms such as nausea, dizziness, weakness, and confusion may occur. When the concentrations exceed 3.5 mmol/L, more pronounced neurological manifestations occur [34]. Severe hypermagnesemia (typically defined as serum magnesium > 2.0–2.5 mmol/L) is uncommon in hemodialysis patients. Studies indicate that its prevalence is low, often 0–2% in stable chronic hemodialysis populations, although mild hypermagnesemia (>1.5 mmol/L) occurs in 15–20% of patients [35].

Previous studies on magnesium therapy have reported asymptomatic increases in serum magnesium, with peak values around 1.9–2.0 mmol/L and no clinically significant toxicity [26]. However, it is important for clinicians to recognize that sevelamer use is also associated with higher serum magnesium levels.

In our study, during the follow-up period, there was a trend toward increasing magnesium concentrations in both patient cohorts, with a statistically significant increase documented in each group by the end of the treatment. In addition, the proportion of patients with abnormally elevated serum magnesium levels in the magnesium carbonate group increased by only 11%. Nevertheless, no significant between-group differences in serum magnesium levels were observed at any time point, and no clinically apparent side effects were observed in any of the patients. Our study showed that low doses of magnesium carbonate hardly increased the magnesium concentration, confirming its safety and reliability.

Another important aspect of the overall evaluation of phosphate binders is the occurrence of adverse effects (particularly gastrointestinal intolerance), which further contributes to poor adherence to medication. Higher prescribed doses are associated with reduced patient compliance. The dosing regimen is frequently not followed, which decreases the actual amount of medication taken, resulting in poor tolerability and high dropout rates. We administered magnesium carbonate at low doses, which may explain the minimal incidence of adverse effects.

In addition to the absence of adverse effects, a low pill burden further promotes patient adherence. A systematic review found that phosphate binder non-adherence ranged from 13.9% to 98.6%, averaging 52.5% [36]. Key factors affecting non-adherence included pill burden, number of prescribed binders, regimen complexity, and cost of treatment. Only 7% of patients taking more than six tablets daily maintained moderate adherence [37]. In our dialysis center, we have observed the same phenomenon, with treatment with sevelamer being a suitable example. This fact, as well as financial considerations, was the main driver for conducting this study. Subsequently, all patients adhered to the prescribed regimen, which is likely attributable to the lower medication doses used in both groups of patients.

Finally, in medicine, the treatment effect is of primary importance. However, price cannot be ignored. In the CALMAG study, the costs of calcium acetate/magnesium carbonate were compared with those of sevelamer. Sevelamer carbonate costs approximately €1441.75 per patient annually (at a 4800 mg daily dose), whereas calcium acetate/magnesium carbonate costs approximately €172–689 annually [18]. Sevelamer costs 2.1–8.4 times more than magnesium carbonate-based therapy, representing an additional €752–€1270 per patient annually. Ossareh et al. state that in many countries, sevelamer is not covered by insurance, and it may be impossible for the majority of patients to obtain it [38]. This economic burden, which occurs in our context, may also affect its extensive use according to the guidelines. Our data are consistent with those of other studies, as sevelamer carbonate clearly demonstrates higher treatment costs than magnesium carbonate across all periods (per day, per month, and per year).

## 5. Limitations

This study had several limitations. The sample size was relatively small, and lower medication doses were used compared to the standard doses applied in previous studies, particularly those that included calcium and magnesium salt formulations. Moreover, the short follow-up period limited the assessment of long-term outcomes. As a pilot, hypothesis-generating study, no formal power calculation was performed, and the findings were exploratory and intended to guide future larger studies with appropriate statistical adjustments.

## 6. Conclusions

Low-dose magnesium carbonate demonstrated a phosphorus-lowering efficacy that was not inferior to that of low-dose sevelamer in patients undergoing chronic hemodialysis while maintaining stable calcium–phosphorus parameters and good tolerability. Given its favorable safety profile, absence of gastrointestinal side effects, and lower cost, magnesium carbonate has emerged as a promising phosphate binder option that could help address pill burden and compliance challenges in routine practice. It is important to increase physicians’ awareness of the clinical and economic benefits of magnesium carbonate as a potential first-line phosphate management strategy. Larger prospective studies across diverse dialysis centers are warranted to further establish the long-term efficacy, safety, and real-world applicability of magnesium carbonate in routine nephrology practice.

## Figures and Tables

**Table 1:** Comparison of groups’ laboratory parameters measured before treatment initiation.

Parameter	Magnesium Carbonate (*n* = 29)	Sevelamer Carbonate (*n* = 25)	*p*
*Demographic characteristics*			
Age (Mean ± SD)			
	55.9 ± 13.5	61.5 ± 10.9	*p* = 0.105
Sex %(*n*)			
*male*	51.7% (19)	52% (6)	*p* = 0.600
*female*	48.3% (5)	58% (13)	
*Laboratory parameters (Mean ± SD)*			
RBC	4.2 ± 1.6	4.1 ± 1.4	*p* = 0.81
HGB	123 ± 12.7	121 ± 11.4	*p* = 0.54
PLT	296 ± 78.9	320 ± 86.4	*p* = 0.29
WBC	8.3 ± 3.4	7.9 ± 2.1	*p* = 0.61
Albumin	41 ± 4.3	43 ± 5.7	*p* = 0.15
Phosphorus (mmol/L)	2.32 ± 0.35	2.18 ± 0.37	*p* = 0.179
Calcium (mmol/L)	2.26 ± 0.24	2.33 ± 0.24	*p* = 0.298
Magnesium (mmol/L)	0.72 ± 0.08	0.88 ± 0.11	*p* < 0.001
PTH (pg/mL)	343.18 ± 273.90	488.96 ± 322.81	*p* = 0.078

**Table 2:** Comparison of the therapy parameters at baseline and end of study in the Magnesium carbonate group using a paired *t*-test.

Indicator (Parameter and Month)					Paired Difference				
		Mean	SD	Percent Change	95% Confidence Interval of the Difference		t	df	Sig. (2-Tailed)
					Lower	Upper			
1	P_0—P_3	0.326	0.351	−14.05%	0.190	0.462	4.926	27	0.000
2	Ca_0—Ca_3	−0.023	0.304	+1.02%	−0.141	0.094	−0.416	27	0.681
3	Mg_0—Mg_3	−0.224	0.151	+31.11%	−0.283	−0.165	−7.836	27	0.000
4	PTH_0—PTH_3	−54.669	186.548	+15.93%	−125.628	16.290	−1.578	28	0.126

Abb: P_0—Phosphorus at month 0; P_3—Phosphorus at month 3; Ca_0—Calcium at month 0; Ca_3—Calcium at month 3; Mg_0—Magnesium at month 0; Mg_3—Magnesium at month 3; PTH_0—Parathyroid hormone at month 0; PTH_3—Parathyroid hormone at month 3.

**Table 3:** Comparison of the Effect of Therapy at Baseline and End of Study in the Sevelamer carbonate group.

Indicator (Parameter and Month)					Paired Difference				
		Mean	SD	Percent Change	95% Confidence Interval of the Difference		t	df	Sig. (2-Tailed)
					Lower	Upper			
1	P_0—P_3	0.111	0.529	−5.09%	−0.107	0.329	1.051	24	0.304
2	Ca_0—Ca_3	0.121	0.264	−5.19%	0.011	0.230	2.290	24	0.031
3	Mg_0—Mg_3	−0.062	0.101	+7.05%	−0.104	−0.020	−3.097	24	0.005
4	PTH_0—PTH_3	123.168	476.898	−25.19%	−73.686	320.022	1.291	24	0.209

Abb: P_0—Phosphorus at month 0; P_3—Phosphorus at month 3; Ca_0—Calcium at month 0; Ca_3—Calcium at month 3; Mg_0—Magnesium at month 0; Mg_3—Magnesium at month 3; PTH_0—Parathyroid hormone at month 0; PTH_3—Parathyroid hormone at month 3.

**Table 4:** Results of the independent samples *t*-tests comparing the two treatment groups at each time point.

Parameter		Mean	SD	95% Confidence Interval for Mean		t	df	Sig.
				Lower Bound	Upper Bound			
P_1	magnesium carbonate	2.182	0.540	1.977	2.387	2.663	52	0.010
	sevelamer carbonate	1.861	0.289	1.742	1.980			
Ca_1	magnesium carbonate	2.217	0.172	2.151	2.282	2.170	52	0.035
	sevelamer carbonate	2.095	0.239	1.997	2.194			
Mg_1	magnesium carbonate	0.981	0.188	0.909	1.052	−1.058	52	0.295
	sevelamer carbonate	0.832	0.183	0.782	0.882			
P_2	magnesium carbonate	2.100	0.564	1.885	2.315	0.932	52	0.356
	sevelamer carbonate	1.968	0.457	1.780	2.157			
Ca_2	magnesium carbonate	2.210	0.156	2.151	2.269	−0.257	52	0.798
	sevelamer carbonate	2.225	0.271	2.113	2.337			
Mg_2	magnesium carbonate	0.972	0.156	0.912	1.031	1.143	52	0.258
	sevelamer carbonate	0.928	0.119	0.879	0.977			
P_3	magnesium carbonate	2.014	0.454	1.838	2.190	−0.486	51	0.629
	sevelamer carbonate	2.072	0.423	1.898	2.247			
Ca_3	magnesium carbonate	2.286	0.136	2.233	2.339	1.434	51	0.158
	sevelamer carbonate	2.212	0.231	2.117	2.308			
Mg_3	magnesium carbonate	0.956	0.158	0.894	1.017	0.268	51	0.789
	sevelamer carbonate	0.945	0.122	0.895	0.995			
PTH_3	magnesium carbonate	410.350	297.012	295.181	525.519	0.412	51	0.682
	sevelamer carbonate	365.800	478.230	168.396	563.204			

**Table 5:** Comparison of the adverse effects, pill burden, and cost of low-dose sevelamer vs. low-dose magnesium carbonate.

	*Sevelamer Carbonate* (*n* = 25)	*Magnesium Carbonate* (*n* = 29)
***Adverse effects***		
GI intolerability	1	1
Other side effects	0	0
***Pill burden and economic comparison***		
Milligrams per tablet/Tablets per package	800 mg/180	250 mg/30
Package price (€)	59.42 €	5.01 €
Cost per tablet (€)	0.33 €	0.17 €
Cost per day (**€**)	≈€2.97	≈€0.51
Cost per month (**€**)	≈€89.10	≈€15.30
Cost per year (**€**)	≈€1084.05	≈€186.15
Pill burden	3	1

The prices of the medications correspond to those in the national pharmacy network and are up to date as of the time the study was conducted.

## Data Availability

The raw data supporting the conclusions of this article will be made available by the authors on request.

## Abbreviations

The following abbreviations are used in this manuscript:

Ca	Calcium
Ca × P	Calcium-Phosphorus product
CKD	Chronic Kidney Disease
CKD-MBD	Chronic Kidney Disease-Mineral and Bone Disorder
EAD	Bulgarian Solely Owned Joint Stock Company
ESRD	End-Stage Renal Disease
FGF23	Fibroblast Growth Factor 23
KDOQI	Kidney Disease Outcomes Quality Initiative
KDIGO	Kidney Disease: Improving Global Outcomes
Mg	Magnesium
MgCO_3_	Magnesium carbonate
MS	Microsoft
P	Phosphate
PTH	Parathyroid hormone
SD	Standard deviation
